# Impact of position of bolster on outcome of prone PCNL—A randomized clinical trial

**DOI:** 10.1002/bco2.457

**Published:** 2024-12-18

**Authors:** Vikram Singh, Ishwar Ram Dhayal, Sanjeet Kumar Singh, Alok Srivastava, Nandan Rai

**Affiliations:** ^1^ Department of urology and renal transplant Dr. Ram Manohar Lohia institute of medical sciences Lucknow India

**Keywords:** bolster, PCNL, pleural, superior calyceal puncture, supracostal puncture, complication

## Abstract

**Objective:**

To determine whether the position of the bolster affects the access tract (supracostal/infracostal) for a superior calyceal puncture during prone PCNL and its effect on pleural complications.

**Materials and Methods:**

It was a randomized clinical trial. Patients in whom superior calyceal puncture was done were divided into two groups by systematic sampling method, group 1 (horizontal bolster) and group 2 (vertical bolster), 50 patients in each group. Standard PCNL was perfomed in all patients. Chest x‐ray was done on POD 0 (postoperative day) and POD 1 for assessment of pleural complication. NCCT KUB was done on POD 1 for assessment of stone clearance.

**Results:**

In group 1, 36 patients (72%) underwent supracostal puncture and 14 patients (28%) underwent infracostal puncture while in group 2, 38 patients (76%) underwent supracostal puncture and 14 patients (28%) underwent infracostal puncture (p‐value‐ 0.820). Two patients (4%) in group 1 & three patients in group 2 had pleural complications in the form of hydrothorax (p‐value‐ 0.666). Four patients in group 1 and five patients in group 2 underwent ancillary procedure for clearance of residual stones.

**Conclusion:**

In our study, the orientation of the bolster either horizontal or vertical does not affect the site of puncture during prone PCNL which probably resulted in no difference in pleural complications in two groups.

## 1 INTRODUCTION

Nephrolithiasis is a global problem that affects all regions of the world.^1^ Ever since Fernstorm and Johansson initially reported on the removal of kidney stones via nephrostomy, PCNL has emerged as the most widely utilized kidney stone treatment technique.^2^ Many changes and adjustments have been made to lower morbidity, analgesic requirements and hospital stays, including the use of regional blocks, single‐stage dilatation, the “mini‐perc” method, tubeless PCNL and sandwich therapy.^3^, ^4^, ^5^ Despite improvements in endoscopic technology and PCNL procedures, surgery is still associated with a high risk of complications, of which injury to the pleura or abdominal viscera is the most concerning.

In a supracostal approach, the risk of intrathoracic problems increases significantly.^6^ To allow expansion of the chest and abdominal wall during PCNL in the prone position, rolled blankets or foam/gel pads are positioned. They can be placed horizontally across the symphysis pubis and lower ribcage or vertically along the lateral parts of the ribcage. The orientation of the cushions varies considerably between centres performing PCNL in the prone position. To date, no evaluation of optimal pillow orientation to maximize kidney position during prone PCNL has been performed.

The main aim of using bolsters in prone PCNL is to provide some room to the abdomen during breathing. This prevents compression of the abdomen, which can worsen diaphragmatic movement and increase pressure in the airways. The bolster is positioned for a second reason: it supports the bony prominences, which allows for better spinal alignment and creates an opening between the iliac crest and the rib cage, which is the perfect place to insert a PCNL access needle. Recent research has suggested that the kidney may shift caudally in a horizontal bolster position, which may reduce the risk of pleural complications during supracostal puncture and superior calyceal puncture.^7^, ^8^

However, the above observations were obtained from voluntary subjects which were imaged using bolster in horizontal and vertical positions. As far as we know, further research on patients is still required to assess the aforementioned finding. We conducted this study in patients undergoing prone PCNL to assess the impact of the orientation of bolster on access (supracostal/infracostal) during superior calyceal punctures and to increase our knowledge of whether we can decrease the need for supracostal punctures while targeting the superior calyx for maximal stone clearance.

## 2 PRIMARY OBJECTIVE

To determine the effect of the position of the bolster on puncture (supracostal/infracostal) during prone PCNL.

## 3 SECONDARY OBJECTIVE

To assess if there is variation in pleural complications with bolster position (horizontal vs vertical).

## 4 MATERIAL AND METHODS

This was a Randomized clinical trial for which the approval of the institution's ethics committee was obtained. The study was registered in the Clinical Trials Registry of India (registration number: CTRI/2023/04/051613).

**FIGURE 1:**
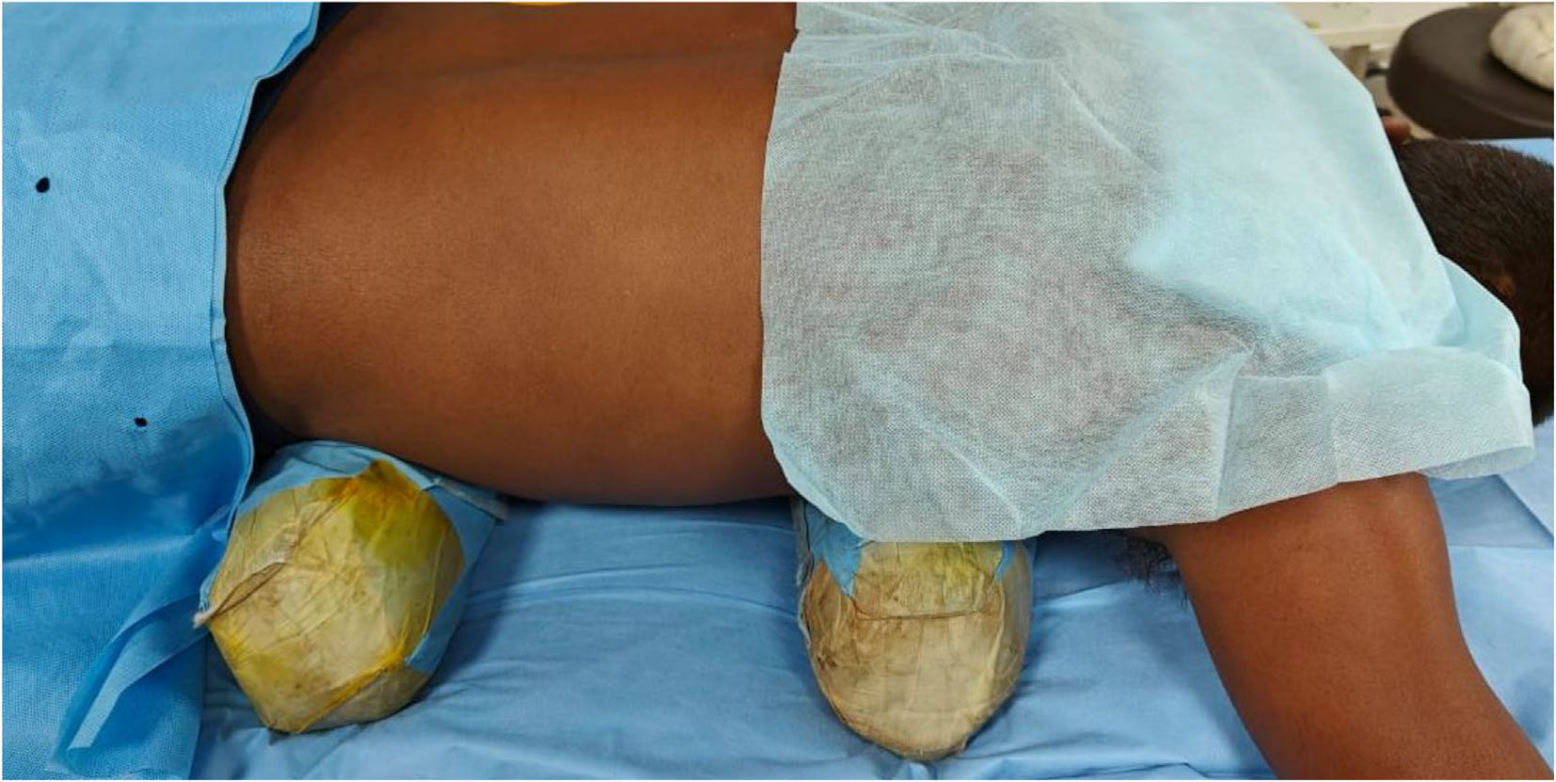
Demonstration of horizontal placement of bolster.

**FIGURE 2:**
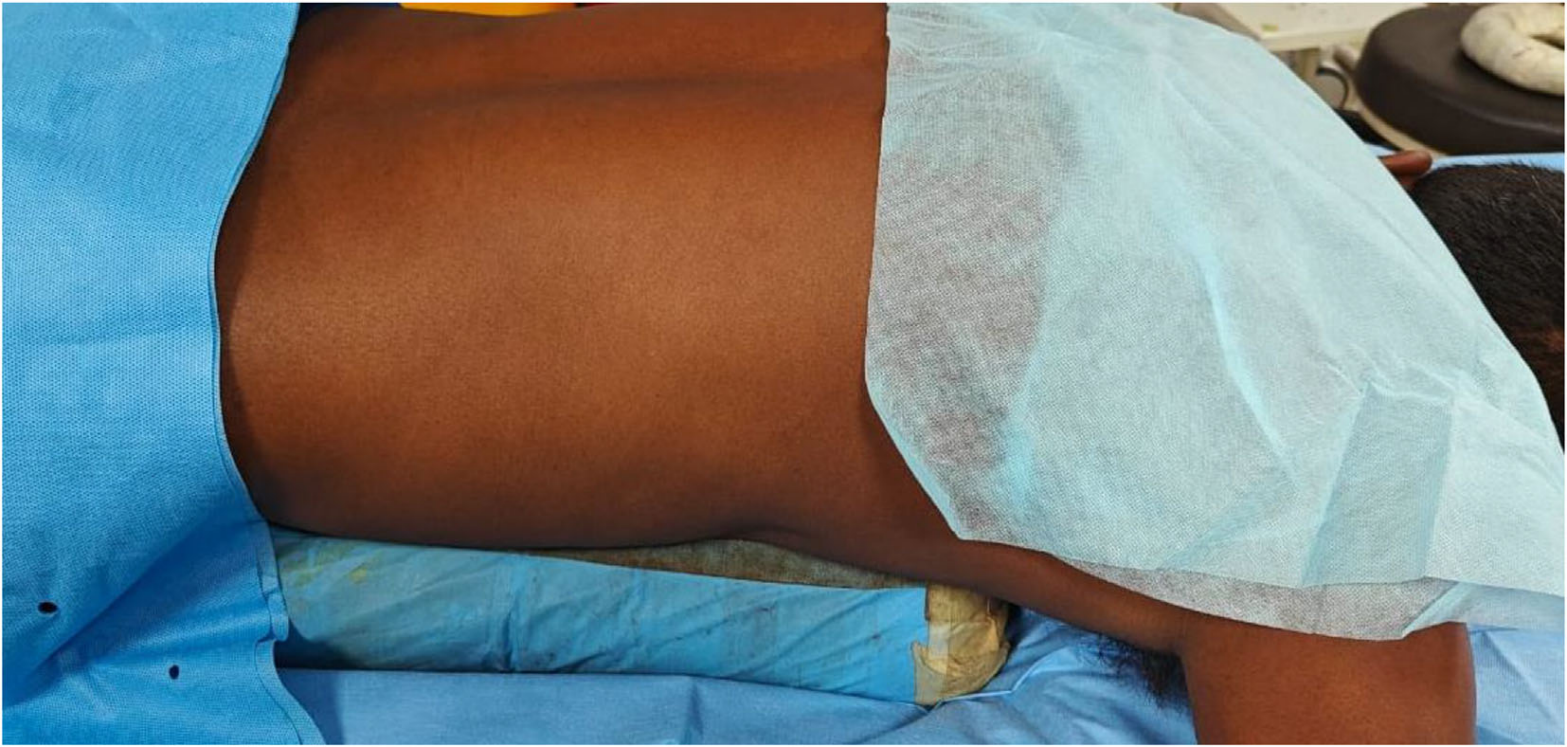
Demonstration of vertical placement of bolster.

**FIGURE 3:**
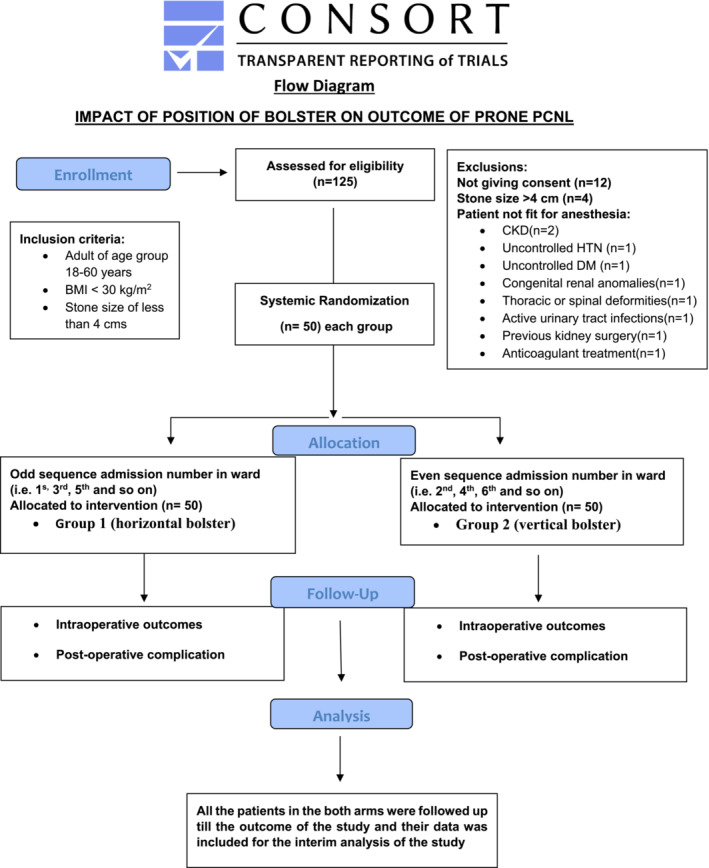
Showing schematic plan of the study.

The institutional ethics committee gave its approval for this randomized clinical trial. The Clinical Trials Registry of India registered the study (registration number: CTRI/2023/04/051613). After evaluation of the preoperative CT urogram, patients who met the inclusion criteria and were scheduled for superior calyceal puncture were divided into two groups depending on whether the pillows were placed horizontally or vertically in the prone position during PCNL as demonstrated in the Figures 1 and 2. In our department, the study was conducted from August 2022 to July 2023. Using a two‐sided test with a significance level of 5% and a power of 90%, the sample size for the two independent groups was set at 100 and 50 in each group, respectively. Systematic sampling method was used for randomization where the odd sequence admission number in ward administered horizontal bolster and allocated to group 1 and the patient with even sequence in ward was given vertical bolster and allocated to group 2 (Figure 3 consort flow diagram). The consort checklist for the article has been added in the supplementary material.

Patient demographics, medical history, clinical examination, relevant blood profile (CBC, viral markers, BUN and serum creatinine), urine tests (routine and culture) and preoperative blood tests were collected.

The study included patients with a BMI of less than 30, an age between 18 and 60 years and a stone size of less than 4 cm. Patients with uncontrolled hypertension or diabetes, chronic kidney disease, congenital renal anomalies, thoracic or spinal deformities, active urinary tract infections, previous kidney surgery, anticoagulant treatment or who were not suitable for general anaesthesia were excluded.

The patient was placed in the prone position with (horizontal/vertical) pillows; foam pillows measuring 50x18x18 cm were used. The vertical pillows were positioned along the lateral chest wall on both sides, while the horizontal pillows were positioned superiorly at the xiphoid process and inferiorly at the pubic bone. PCNL was performed in the prone position, placing the bolsters in both directions, the effects on the puncture site (supracostal/infracostal) while targeting the superior calyx was studied.

Surgical steps: After implantation of a 6‐Fr ureteral catheter and retrograde pyelography, a fluoroscopy‐guided puncture (also known as “needle eye” or “porthole”) was performed to puncture the superior calyx. A 0.035‐in. hydrophilic guidewire with a flexible tip was inserted into the ureter with the first puncture needle. After positioning a 10 Fr Alken metal cannula over the guidewire, a PTFE Amplatz dilator was used to dilate the area in one step. A 24 Fr/26 Fr Amplatz sheath was then slide over the dilator. The stones were fragmented with a pneumatic lithotripter and nephroscopy was performed. Once the stones were removed endoscopically and fluoroscopically, a 6/26 Fr double J stent was inserted.

A chest X‐ray was performed on day 0 after surgery. On the first postoperative day, a chest X‐ray and an X‐ray KUB were performed to identify any pleural problems and to remove any stones.

## 5 STATISTICAL ANALYSIS

Categorical variables were presented as number and percentage (%) and continuous variables as mean and SD. Quantitative variables were compared between two groups using an independent t‐test. Qualitative variables were compared using the chi‐square test or Fisher's exact test. A p‐value of < 0.05 with a confidence interval of 95% was considered statistically significant. Data analysis was performed using the Statistical Package for Social Sciences (SPSS) version 23.0.

## 6 RESULTS

Between August 2022 and July 2023, 100 patients underwent PCNL in the prone position with superior calyceal puncture. Patients were alternately divided into two groups. Fifty patients had the cushion placed in a horizontal position (group 1) and fifty patients in a vertical position (group 2). The preoperative demographic parameters were statistically similar in both groups (Table 1). The average stone size in group 1 was 22.3 mm and in group 2 22.8 mm. In group 1, 36 patients (72%) underwent puncture above the 12th rib and 14 patients (28%) underwent infracostal puncture for superior calyceal access. In group 2, 37 patients (74%) underwent a supra 12th rib puncture, 1 patient required a supra 11th rib puncture and 14 patients (28%) underwent an infracostal puncture for superior calyceal access. In one patient in group 2, the procedure was aborted due to intraoperative bleeding and a repeat operation was performed after 2 days to remove the remaining stones. The operation time was measured from calyx puncture to placement of the double‐J stent. The average operation time from puncture of the upper calyx to removal of the stone was 47.93 + 19.89 minutes in group 1 and 50.83 + 23.53 minutes in group 2 (p‐value ‐ 0.551) (Table 2). Two patients (4%) in group 1 experienced a pleural complication in the form of hydrothorax, with one patient requiring placement of a chest tube and the other patient being treated conservatively. Three patients in group 2 had a pleural complication, two patients had to have a chest tube inserted and one patient was treated conservatively. A nephrostomy tube was placed in all patients, which was removed on postoperative day 2, except for the patients with pleural complications. In patients with ROS on the postoperative X‐ray KUB, an NCCT‐KUB was performed to document the size of the ROS. Small ROS, which were considered to be less than 4 mm in size and clinically insignificant in the study, were found in three patients in group 1 and four patients in group 2. No intervention was performed in these patients. ROS larger than 4 mm were found in four patients in group 1 and five patients in group 2. Additional intervention was required in these patients. Two patients in group 1 underwent ESWL and two patients required repeat PCNL. In group 2, two patients underwent ESWL and three patients underwent re‐look PCNL to achieve complete clearance (Table 3). Patients with radiolucent stones underwent NCCT KUB on day 1 after surgery to remove the stone.

**TABLE 1:** Patient demographics and pre‐operative stone characteristics.

	Characteristics	Group 1 (horizontal bolster)	Group 2 (vertical bolster)	Total
**Total no. of patients in study**	No. of patients	50	50	100
**Age**	Mean age (mean ± SD) in years	39.79 ± 12.97	41.10 ± 14.20	p‐value‐ 0.635
**Sex distribution**	Male	33	35	p‐value‐ **0.830**
	Female	17	15	
**Laterality**	Right	27	28	p‐value‐ 0.841
	Left	23	22	
**Stone size**	10–20 mm	23	21	p‐value‐ 0.501
	20–30 mm	17	14	
	30–40 mm	10	15	
	Mean stone size	22.3 mm	21.8 mm	
**Stone opacity**	Radio‐opaque	44 (88%)	42 (84%)	p‐value‐ 0.950
	Radiolucent	6 (12%)	8 (16)	
**Stone location**	Staghorn	2	3	p‐value‐ 0.320
	Partial staghorn	11	8	
	Pelvis	20	18	
	Upper ureter	11	13	
	Lower calyx	6	8	

**TABLE 2:** Intra‐operative outcomes.

	Characteristics		Group 1 (horizontal bolster)	Group 2 (vertical bolster)	Total
**Access site**	Supracostal 12th		36	37	p‐value‐ 0.820
	Supra 11th		0	1	
	Infracostal		14	12	
**Operative time**	Mean (in minutes)		47.93 ± 19.89	50.83 ± 23.53	p value‐ 0.551
**Stone clearance**	Complete		43 (86%)	41 (82%)	p‐value‐ 1.00
	Small ROS (<4 mm)		3	4	
	ROS (>4 mm)		4	5	
**Comparison between Right and Left kidney**	Right	Supracostal	20	24	p‐value‐ 0.841
		Infracostal	7	4	
	Left	Supracostal	15	13	
		Infracostal	8	9	

**TABLE 3:** Post‐operative complications and ancillary procedure.

	Characteristics	Group 1 (horizontal bolster)	Group 2 (vertical bolster)	Total
**Pulmonary complication**	Hydrothorax	2 (4%)	3 (6%)	p‐value‐ 1.00
**Complications as per Clavien Dindo classification**	*Grade 1*			p‐value‐ 0.894
	Transient Fever (>100 F)	6	7	
	Haematuria	6	5	
	Pain (analgesic need on post op day 1)	5	7	
	*Grade 2*			p‐value‐0.770
	Blood transfusion	3 (6%)	4 (8%)	
	Post op ileus	0	1 (2%)	
	Nephrostomy tract urine leak	4 (8%)	4 (8%)	
	*Grade 3a*			p‐value‐ 0.714
	Bacteraemia/sepsis	2 (4%)	1(2%)	
	Pelvi‐calyceal tear/perforation	4 (8%)	4 (8%)	
	Hydrothorax requiring chest tube drain placement	1 (2%)	2 (4%)	
	*Grade 3b, 4a,4b,5*	Nil	Nil	
**Need for ancillary procedure**	ESWL	2 (4%)	2 (4%)	p‐value‐1.00
	Re‐look PCNL	2 (4%)	3 (6%)	p‐value‐0.666

## 7 DISCUSSION

PCNL is the gold standard in the treatment of kidney stones worldwide. Although the procedure is minimally invasive, it is still associated with potential complications and morbidity. To increase the safety of the procedure, a number of modifications are made, such as variations in patient position (prone, supine, modified supine), reducing the size of the access tract (mini, ultra‐mini, micro PCNL) and avoiding supracostal puncture for renal access for fear of pulmonary complications. Upper renal septal puncture is a versatile technique as it provides access to a maximum number of renal cells and allows the urologist to remove a maximum stone burden, especially in cases of staghorn calculi and multiple renal stones. In stones larger than 4 cm the average operative time and number of puncture for stone clearance increases, also there is a significant association of larger stone sizes more than 4 cm with post‐operative SIRS/urosepsis, which can lead to bias in study, hence it was not included in the study. The surgeon can require the supra 12th rib and sometimes the supra 11th rib to gain access to the upper calyx, which can lead to pleural injury and hydrothorax.

There are few recent studies suggesting that bolster alignment can affect the anatomic alignment of the kidney. In a study by Singh et al., it was observed that there is caudal displacement of both kidneys when the bolsters are placed and the displacement is greater when they are placed in the horizontal direction. The craniocaudal displacement of the kidney was measured by the distance between the upper calyx and the diaphragm by placing the kidney roll in horizontal and vertical positions. A mean difference of 2.1 cm was observed for the right kidney (p‐value < 0.01) and 1.5 cm for the left kidney (p‐value < 0.01) between the horizontal and vertical positions of the bolster. These observations were made when the patients were brought to the CT urogram and the bolsters were placed as they would be positioned in the operating room. They suggested that a greater caudal displacement of the upper renal pole by 1–2 cm can result in fewer supracostal punctures being required for superior calyceal access and a decrease in the number of pleural injuries.^7^

A similar study was conducted by Sagalovich et al. in which they examined 10 healthy volunteers with 20 renal units using MRI. They observed that in the right kidney, the mean distance between the kidney and the diaphragm increased from 2.68 cm to 6.12 cm (mean difference ‐ 3.44 cm, p‐value ‐ 0.02) when the renal dish was moved from the vertical to the horizontal position. In the left kidney, the distance between the kidney and the diaphragm increased from 3.54 cm to 5.40 cm (mean difference ‐ 1.84 cm, p‐value ‐ 0.01). With this result, they also suggested that placement of a horizontal pad could reduce the need for supracostal access and improve safety during supracostal access in PCNL.^8^

We performed PCNL in the prone position, placing the bolsters in both directions and studied the effects on the puncture site (supracostal/infracostal) while targeting the superior calyx. All punctures were performed under general anaesthesia with normal expiratory posture. After performing PCNL in the prone position in 100 patients, 36 patients in group 1 underwent supracostal puncture (all supra 12th rib) and 38 patients in group 2 underwent supracostal puncture (37 supra 12th rib and 1 supra 11th rib). Fourteen patients in group 1 and twelve patients in group 2 underwent infracostal puncture. We found that there was no significant difference in the amount of puncture required for the superior calyceal approach in the 2 groups (p‐value‐0.603). Thus, out of 100 patients, 74 patients required supracostal puncture for superior calyceal access. From our results, it can be concluded that there is a 70–75% probability that the patient will require a supracostal puncture if a superior calyceal approach is planned, especially in the case of staghorn calculus and multiple renal calculi. This can also be suspected preoperatively and used for better patient counselling with regard to possible postoperative complications.

In our study, two patients in group 1 (4%) and three patients in group 2 (6%) developed hydrothorax. One patient in group 1 (2%) and two patients in group 2 (4%) required chest tube placement. The difference was not significant (p‐value > 0.05), indicating that the orientation of the bolster has no influence on the pleural complication of PCNL. Supracostal puncture was performed in all patients who developed hydrothorax. Of the 74 patients who underwent supracostal puncture, 5 patients (6.7%) developed a hydrothorax.

### 7.1 Limitations of the study

As the choice of superior calyceal puncture can be limited based on clinician discretion and also varies from local institution to institution protocol, further multi‐centre studies with larger patient populations are needed.

## 8 CONCLUSION

In our knowledge, this is the first study which compares the outcome in prone PCNL surgery using bolster in horizontal and vertical positions. In our study, orientation of bolster either horizontal or vertical does not affect the site of puncture during prone PCNL which probably resulted in no difference in pleural complications in two groups. We performed the study in 100 patients, more studies with larger patient population are required which can give more insight to support our findings and standardize the orientation of bolster in prone PCNL surgery.

## AUTHOR CONTRIBUTIONS

**Alok Srivastava (AS):** Conceptualisation; study design; reviewing manuscript; data collection. **Ishwar Ram Dhayal (IRD):** Conceptualisation; reviewing manuscript; data collection. **Sanjeet Kumar Singh (SKS):** Study design; reviewing manuscript; data collection. **Vikram Singh (VS):** Study design; writing manuscript; data collection. **Nandan Rai (NR):** Writing manuscript; data collection; data analysis.

## CONFLICT OF INTEREST STATEMENT

The authors declare no conflict of interests exists.

## Supporting information


**Data S1.** Supporting Information.
